# Cell death and ultrastructural alterations in *Leishmania amazonensis* caused by new compound 4-Nitrobenzaldehyde thiosemicarbazone derived from *S*-limonene

**DOI:** 10.1186/s12866-014-0236-0

**Published:** 2014-09-26

**Authors:** Elizandra Aparecida Britta, Débora Botura Scariot, Hugo Falzirolli, Tânia Ueda-Nakamura, Cleuza Conceição Silva, Benedito Prado Dias Filho, Redouane Borsali, Celso Vataru Nakamura

**Affiliations:** Programa de Pós-graduação em Ciências Farmacêuticas, Universidade Estadual de Maringá, Avenida Colombo, Jd. Universitário, Maringá, PR 5790 Brazil; Departamento de Química, Universidade Estadual de Maringá, Maringá, PR Brazil; Université Grenoble Alpes, CERMAV-CNRS UPR 5301, Grenoble, F-38000 France

**Keywords:** Antileishmanial activity, Benzaldehyde thiosemicarbazone, *Leishmania amazonensis*, Ultrastructural alterations, Mitochondria damage, Electron microscopy, Cellular disorganization

## Abstract

**Background:**

The treatment of leishmaniasis with pentavalent antimonials is problematic because of their toxicity. Investigations of potentially active molecules are important to discover less toxic drugs that are viable economic alternatives for the treatment of leishmaniasis. Thiosemicarbazones are a group of molecules that are known for their wide versatility and biological activity. In the present study, we examined the antileishmania activity, mechanism of action, and biochemical alterations produced by a novel molecule, 4-nitrobenzaldehyde thiosemicarbazone (BZTS), derived from *S*-limonene against *Leishmania amazonensis*.

**Results:**

BZTS inhibited the growth of the promastigote and axenic amastigote forms, with an IC_50_ of 3.8 and 8.0 μM, respectively. Intracellular amastigotes were inhibited by the compound with an IC_50_ of 7.7 μM. BZTS also had a CC_50_ of 88.8 μM for the macrophage strain J774A1. BZTS altered the shape, size, and ultrastructure of the parasites, including damage to mitochondria, reflected by extensive swelling and disorganization of the inner mitochondrial membrane, intense cytoplasmic vacuolization, and the presence of concentric membrane structures inside the organelle. Cytoplasmic lipid bodies, vesicles inside vacuoles in the flagellar pocket, and enlargement were also observed. BZTS did not induce alterations in the plasma membrane or increase annexin-V fluorescence intensity, indicating no phosphatidylserine exposure. However, it induced the production of mitochondrial superoxide anion radicals.

**Conclusions:**

The present results indicate that BZTS induced dramatic effects on the ultrastructure of *L. amazonensis*, which might be associated with mitochondrial dysfunction and oxidative damage, leading to parasite death.

**Electronic supplementary material:**

The online version of this article (doi:10.1186/s12866-014-0236-0) contains supplementary material, which is available to authorized users.

## Background

Leishmaniasis represents a serious public health problem in 88 countries, occurring mainly in economically disadvantaged regions [1]. Few drug therapy discoveries have been made in recent decades. The first-line treatment for cutaneous leishmaniasis consists of only pentavalent antimonials, but their toxicity is a major issue [2]. Although these drugs are not an ideal treatment, none of the other many investigated drugs are better than pentavalent antimonials with regard to safety, efficacy, and cost [3,4]. Investigations of potentially active molecules are important to discover less toxic drugs that are viable economic alternatives for the treatment of leishmaniasis.

Thiosemicarbazones are a group of molecules whose biological properties have long been investigated [5]. Antiviral, antiprotozoal, antitumor, antimicrobial, and antifungal properties have been reported for some thiosemicarbazones [6,7]. Phase 2 clinical trials that investigated 3-aminopyridine-2-carboxaldehyde thiosemicarbazone, also known as Triapine, were completed for the treatment of metastatic renal cell carcinoma, and patients are currently being recruited for Phase 2 clinical trials for vaginal cancer [8,9].

Thus, this chemical group is known for its wide versatility and biological activity, which is mainly associated with a metal complex or the presence of different substituent groups, such as limonene. These purposeful molecular changes appear to improve the biological properties of the molecule, increase the potential for activity, and lessen cytotoxicity in an attempt to reach a specific target in the parasite, such as mitochondria [10-12]. The main cellular targets of some thiosemicarbazones have been previously reported, indicating from molecular and chemistry targets, such as viral RNA synthesis and tyrosinase activity respectively, to organelles dysfunction, like mitochondria function [13-15]. The present study examined the antileishmania activity and mechanism of action of a novel molecule, 4-nitrobenzaldehyde thiosemicarbazone, derived from *S*-limonene against *L. amazonensis*.

## Results

### BZTS inhibited L. amazonensis proliferation *in vitro*

**Figure 1:**
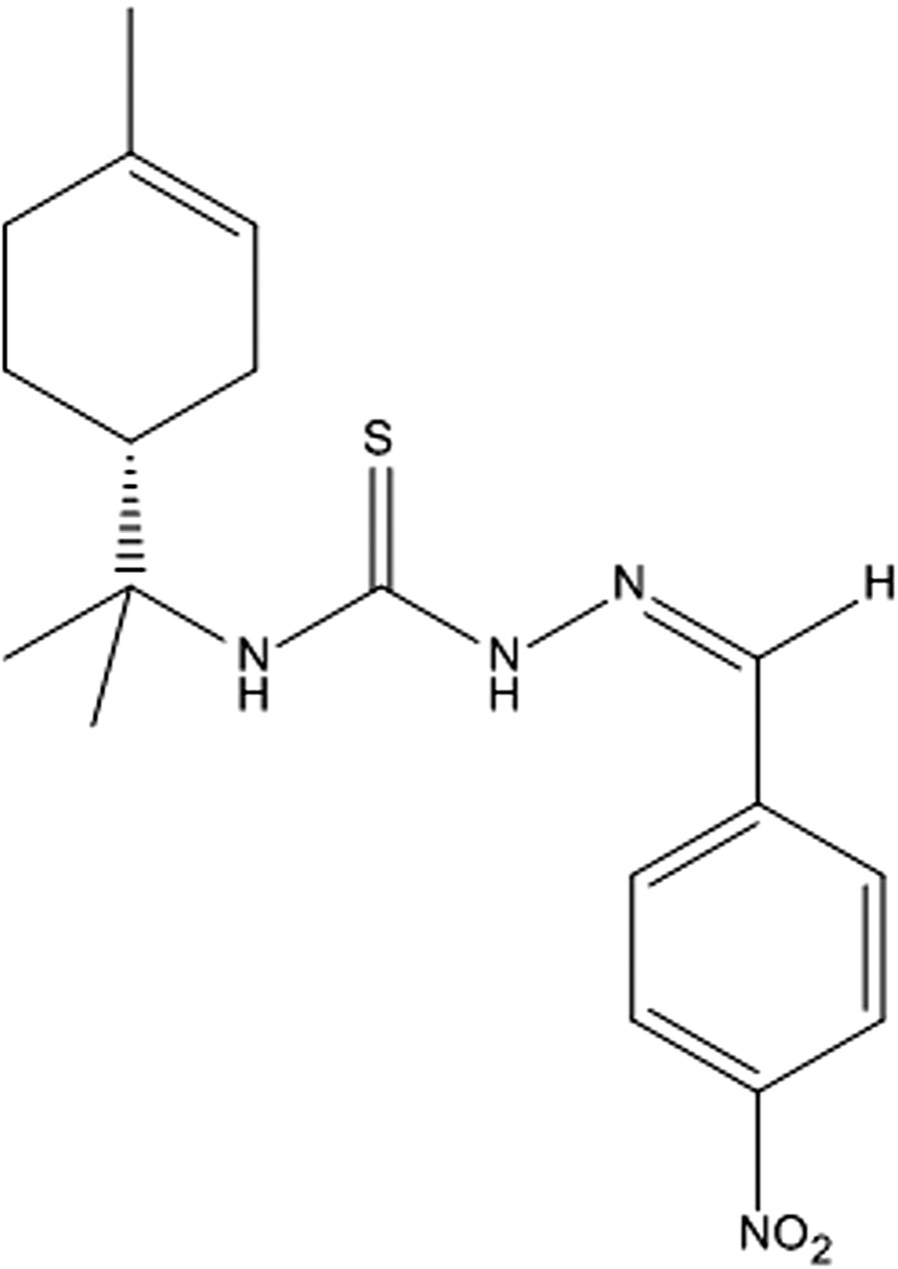
**Structure of 4-nitrobenzaldehyde thiosemicarbazone derived from** ***S*** **-limonene.**

To determine the effects of BZTS (Figure 1) on the cellular proliferation of *L. amazonensis*, the parasites were incubated with BZTS (2.7, 13.9, 27.7, 139 and 277 μM) for 72 h. BZTS dose-dependently inhibited the growth of the promastigote and axenic amastigote forms, with an IC_50_ of 3.8 μM and 8.0 μM, respectively. BZTS exerted activity against intracellular amastigotes at an IC_50_ value of 7.7 μM. BZTS was also evaluated for its cytotoxic effects on the macrophage strain J774A1, with a CC_50_ of 88.8 μM. Cytotoxicity in J774A1 macrophages and activity against promastigotes, axenic amastigotes, and intracellular amastigotes were compared using the selectivity index (SI; ratio: CC_50_ J774A1 macrophage/IC_50_ protozoa). The observed SIs were 23.4, 11.1, and 11.5 for the promastigotes, axenic amastigotes, and intracellular amastigotes, respectively. These results demonstrate that BZTS is more toxic to the protozoa than to macrophages (Additional files 1, 2, and 3).

### BZTS induced morphological and ultrastructural changes

**Figure 2:**
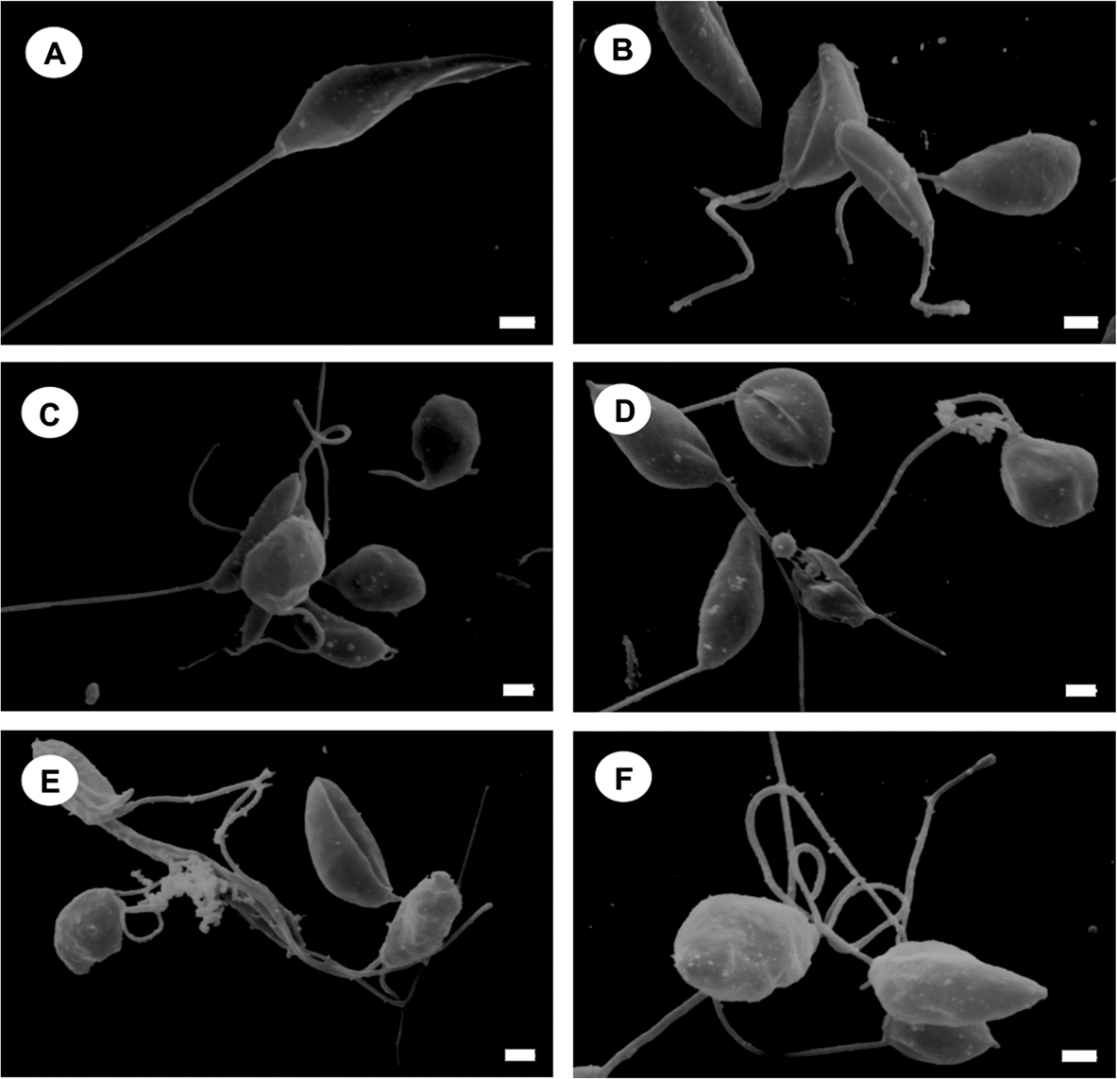
**Scanning electron microscopy of** ***Leishmania amazonensis*** **promastigotes.** The figure shows control promastigotes **(A)** and promastigotes treated with concentrations that corresponded to the IC_50_ **(B-D)** and IC_90_ **(E,F)** of BZTS. **(A)** The protozoan presented typical characteristics, with an elongated shape and free flagellum. **(B-F)** BZTS induced alterations in the shape and size of the protozoan. Scale bar = 1 μm.

**Figure 3:**
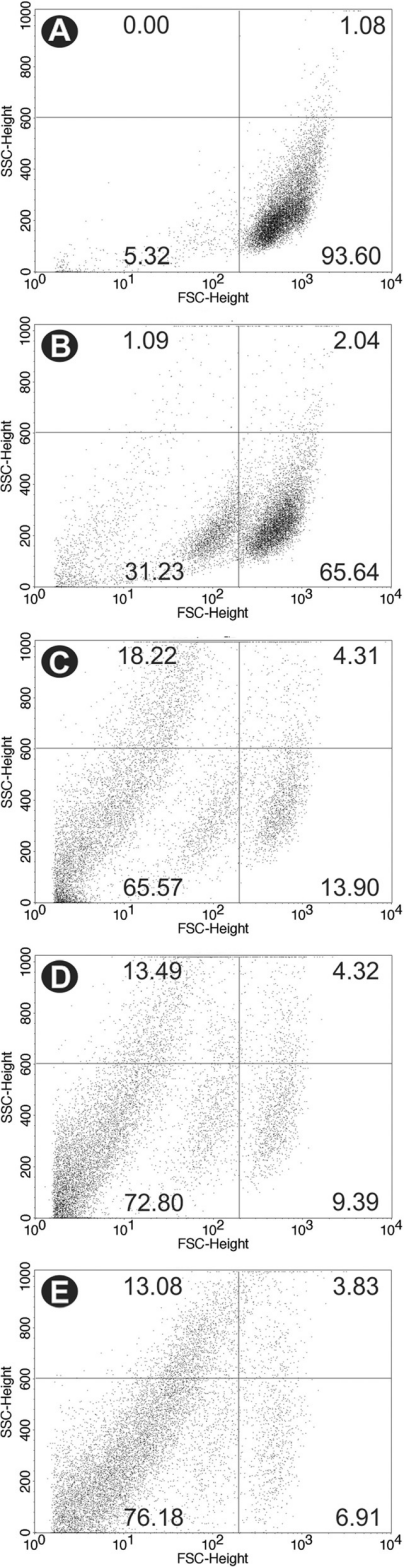
**Cell volume analysis by flow cytometry of untreated** ***Leishmania amazonensis*** **promastigotes (A) and promastigotes treated with 55, 139, 277, and 555** **μM BZTS, respectively (B-E), after 24 h of incubation at 25°C.** SSC-H, side scatter; FSC-H, forward scatter.

Scanning electron microscopy revealed that BZTS caused morphological alterations in the promastigote forms of *L. amazonensis* compared with untreated parasites, which showed typical characteristics, with an elongated shape and free flagellum. Figure 2 shows alterations in shape and size and cellular disintegration in BZTS-treated parasites. These alterations were more pronounced in parasites treated with the IC_90_ (13.9 μM) of BZTS. To evaluate the alterations in cell shape and size in promastigote forms revealed by SEM, the treated parasites were assessed by flow cytometry. The histogram showed that BZTS-treated parasites exhibited a reduction of parasite volume (Figure 3). A dose-dependent decrease in cell volume was observed (32.3%, 83.8%, 86.3%, and 89.3% at BZTS concentrations of 55, 139, 277, and 555 μM, respectively).

**Figure 4:**
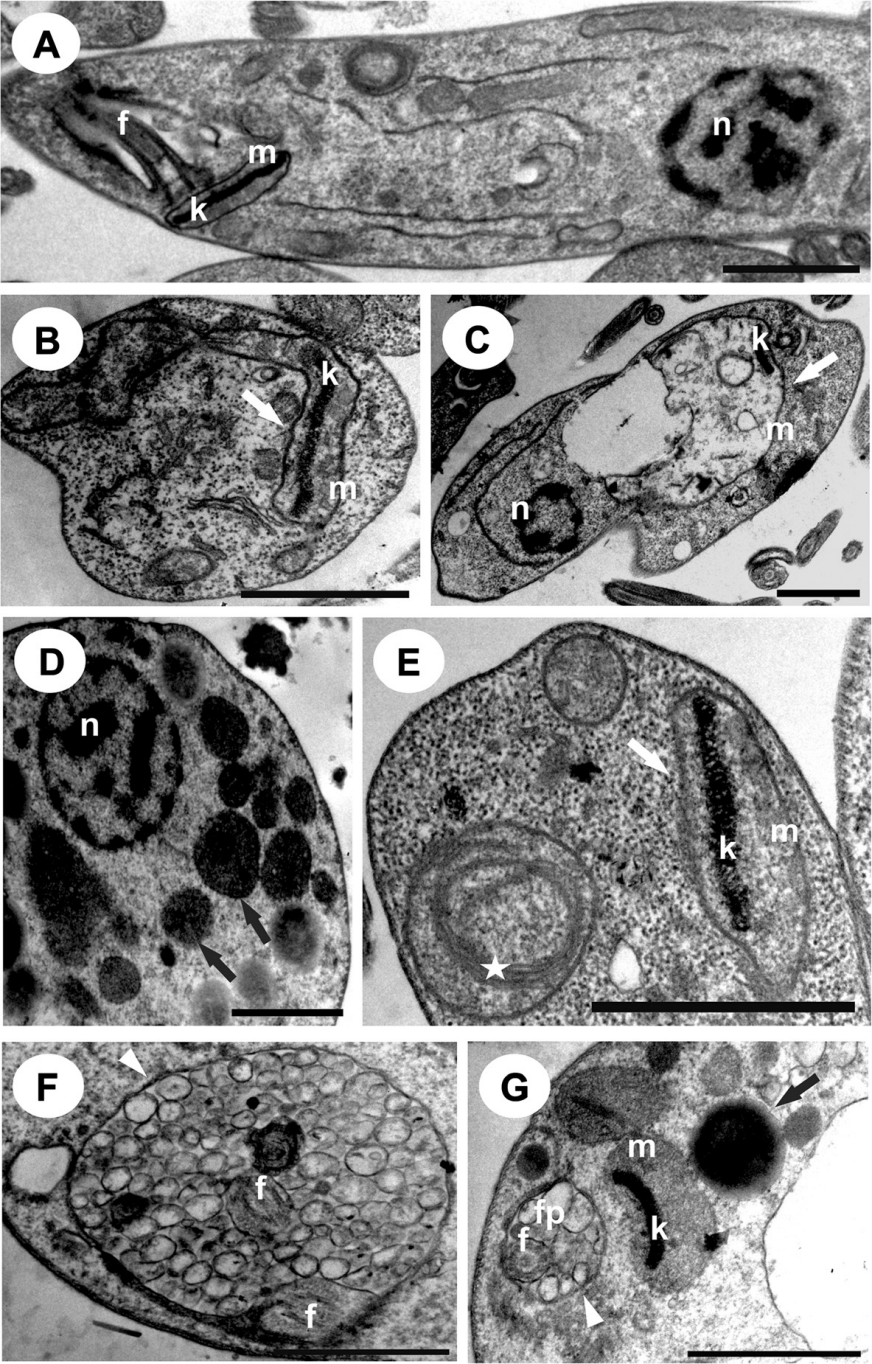
**Ultrathin sections of** ***Leishmania amazonensis*** **promastigotes without treatment that presented a normal ultrastructure (A) and promastigotes treated with BZTS at concentrations that corresponded to the IC**_**50**_ **(B-D) and IC**_**90**_ **(E, G).** White arrows indicate swollen mitochondria. Black arrows represent lipid-storage bodies. White arrowheads indicate the presence of vesicles inside the flagellar pocket. The star indicates the presence of concentric membranous structures. n, nucleus; m, mitochondrion; k, kinetoplast; f, flagellum; fp, flagellar pocket. Scale bar = 1 μm.

**Figure 5:**
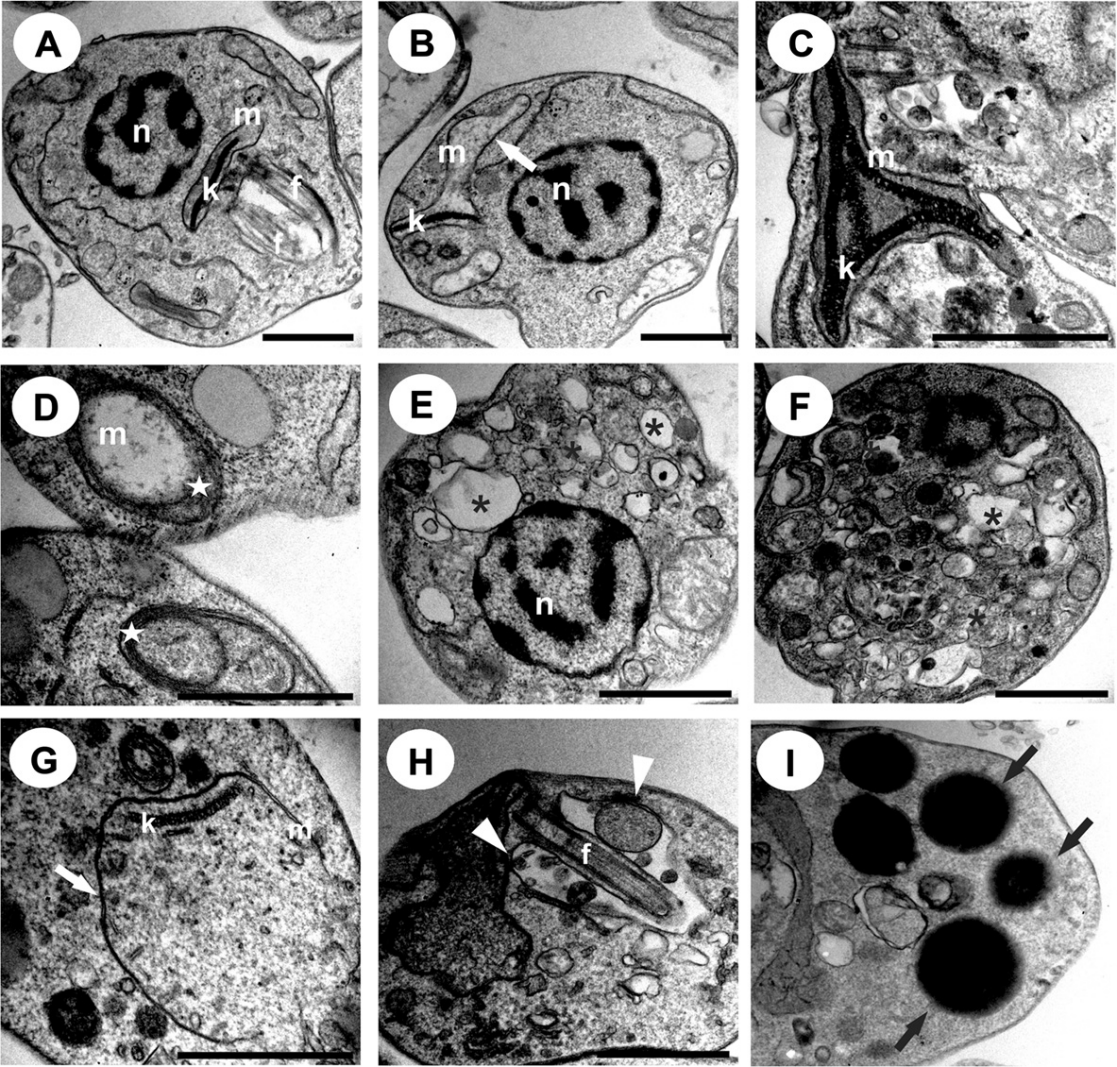
**Ultrathin sections of** ***Leishmania amazonensis*** **axenic amastigotes without treatment that presented a normal ultrastructure (A) and treated with BZTS with concentrations that corresponded to the IC**_**50**_ **(B-F) and IC**_**90**_ **(G,I).** White arrows indicate swollen mitochondria. Black arrows represent lipid-storage bodies. White arrowheads indicate the presence of vesicles inside the flagellar pocket. The star indicates the presence of concentric membranous structures in the mitochondria. The asterisk indicates autophagic vacuoles. n, nucleus; m, mitochondrion; k, kinetoplast; f, flagellum; fp, flagellar pocket. Scale bar = 1 μm.

Transmission electron microscopy of BZTS-treated promastigotes and axenic amastigotes showed the presence of several ultrastructural alterations (Figures 4 and 5). BZTS induced severe damage in parasite mitochondria, reflected by extensive swelling and disorganization in the inner mitochondrial membrane, intense cytoplasmic vacuolization, and the presence of concentric membrane structures inside the organelle. Cytoplasmic lipid bodies, vesicles inside vacuoles in the flagellar pocket, and enlargement were also observed.

**Figure 6:**
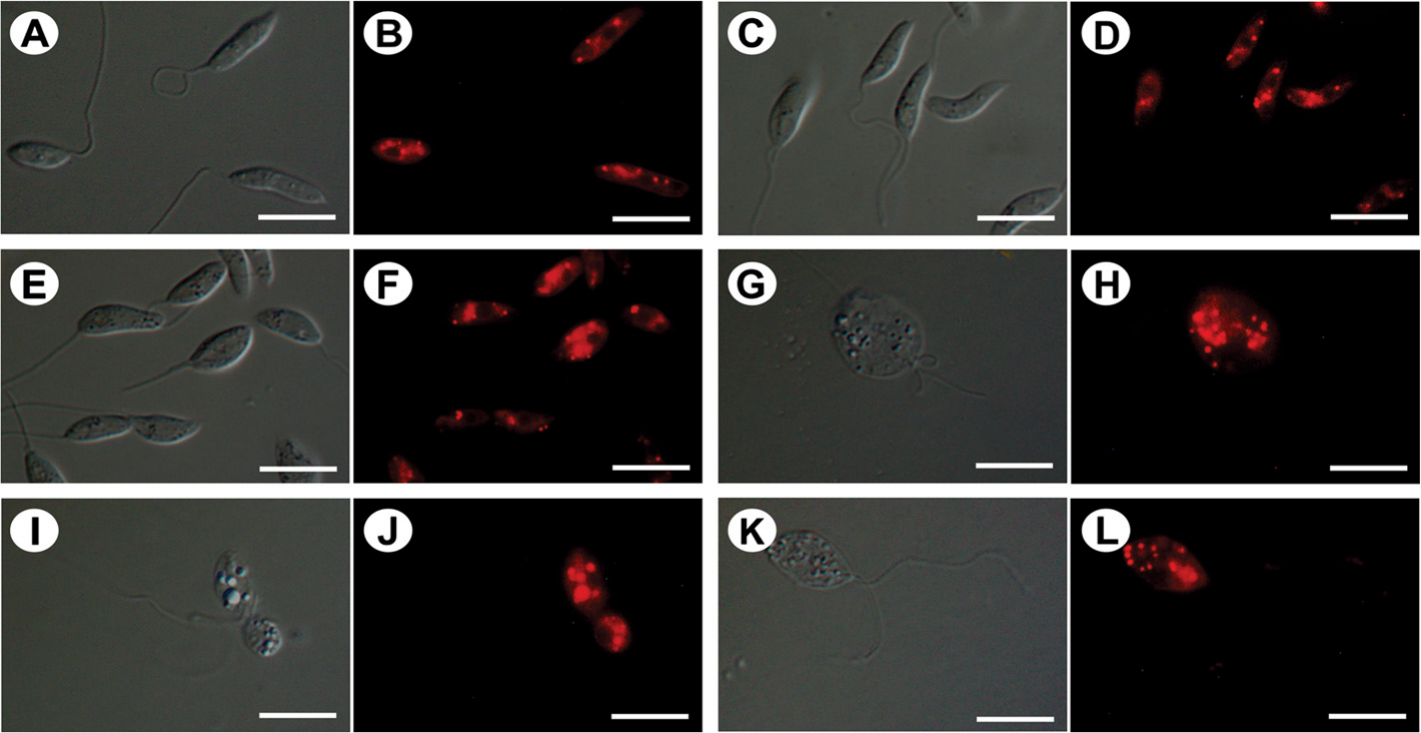
**Differential interference contrast microscopy (DIC) and fluorescence microscopy with Nile Red staining of** ***Leishmania amazonensis*** **promastigotes without treatment (A, B) and treated with concentrations that corresponded to the IC**_**50**_ **(C-F) and IC**_**90**_ **(G-L) of BZTS for 48 h.** In the treated promastigotes, the images suggest the concentration-dependent accumulation of lipid-storage bodies in the cytoplasm. **(G, I, K)** The parasites that were treated with the IC_90_ of BZTS were completely modified. Scale bar = 10 μm.

To confirm the accumulation of lipid bodies in treated promastigotes observed by TEM, we evaluated the existence of this structure using Nile Red staining, which stains neutral lipids. Fluorescence microscopy revealed the presence of lipid bodies in control promastigotes, but the presence of lipid bodies was enhanced after treatment with the IC_50_ and IC_90_ of BZTS for 72 h (Figure 6). This structure appeared to be randomly distributed throughout the parasite cytoplasm.

### BZTS altered ∆Ψm but not cell membrane integrity

**Figure 7:**
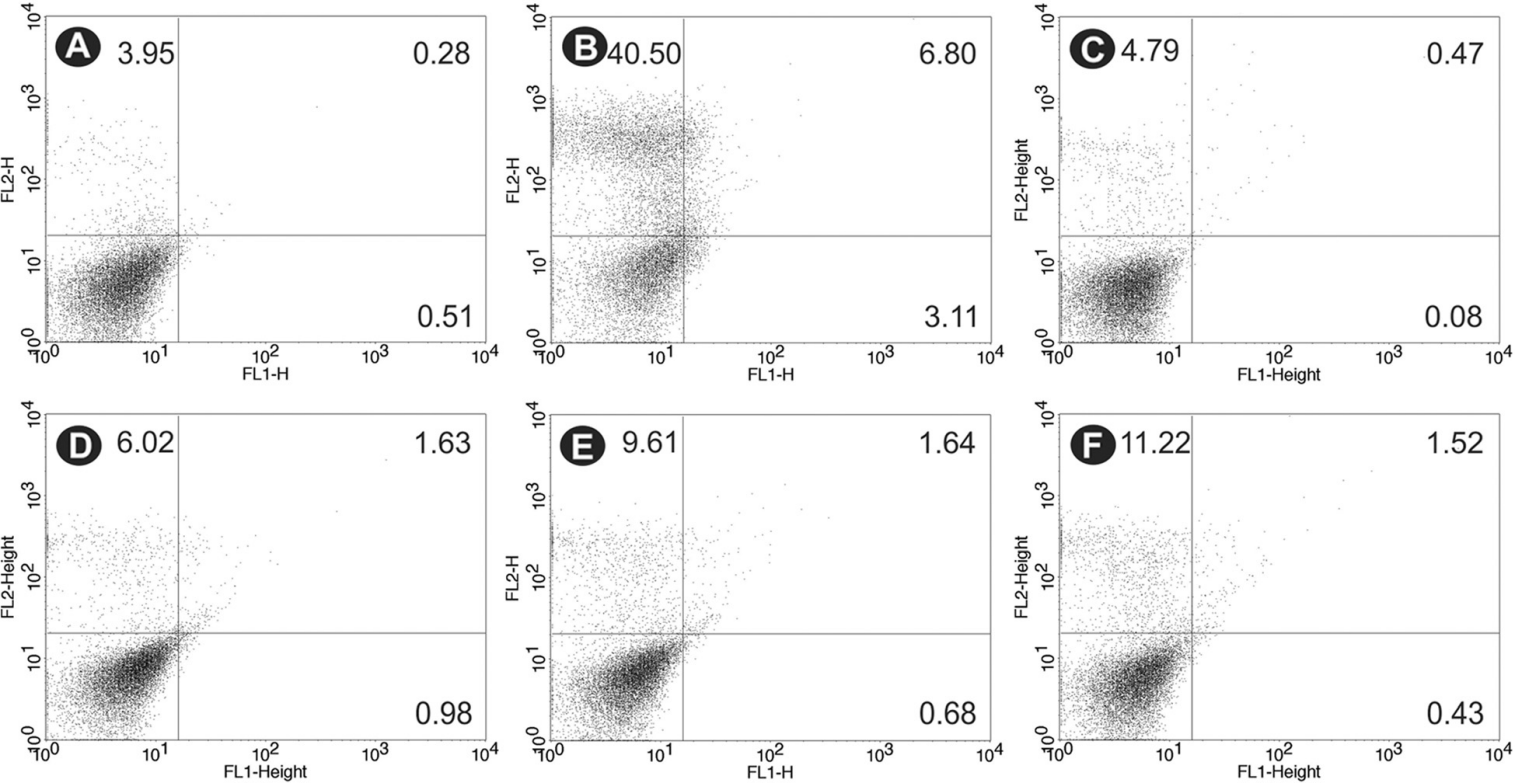
***Leishmania amazonensis*** **promastigotes treated with BZTS and stained with propidium iodide (PI). (A)** Untreated promastigotes. **(B)** Promastigotes treated with 40.0 μM digitonine. **(C-F)** Promastigotes treated with 55, 139, 277, and 555 μM BZTS. The percentage of PI-positive cells is shown in the upper left quadrant.

Alterations in cell membrane integrity were evaluated by PI staining, which is a nucleic acid stain that penetrates cells with a compromised plasma membrane and does not cross the membranes of live cells. BZTS-treated, PI-labeled promastigotes did not show significant permeabilization of the plasma membrane compared with untreated parasites. BZTS at concentrations of 55, 139, 277, and 555 μM showed PI fluorescence intensity of 5.3%, 7.7%, 11.3%, and 12.7%, respectively. The negative control showed PI fluorescence intensity of 4.2%, and the positive control (digitonine) showed PI fluorescence intensity of 47.3% (Figure 7).

**Figure 8:**
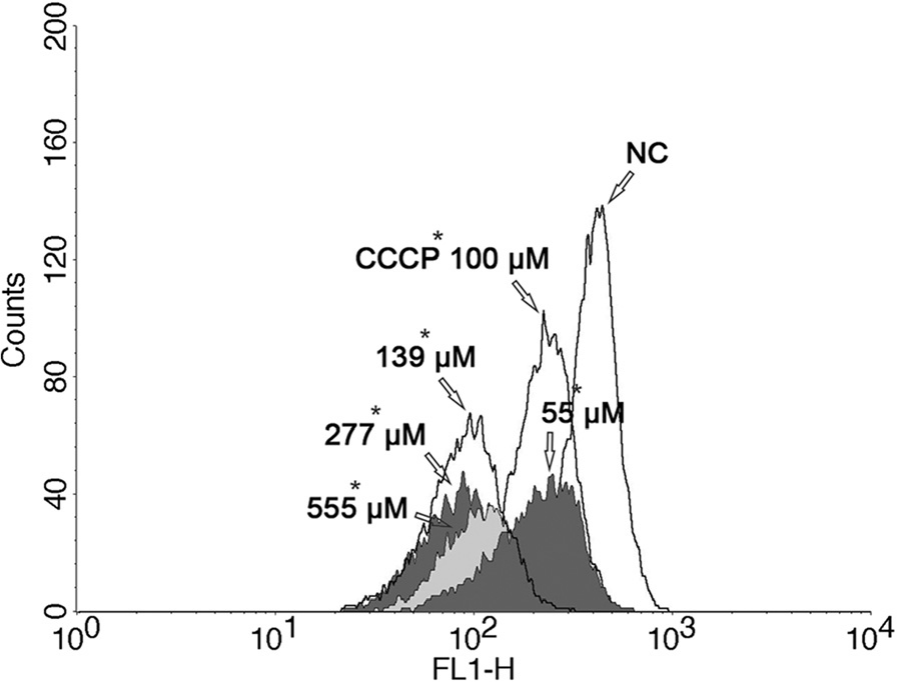
**Rhodamine 123-labeled mitochondrial membrane potential assay by flow cytometry.** The figure shows untreated *Leishmania amazonensis* promastigotes (negative control [NC]), promastigotes treated with 55, 139, 277, and 555 μM BZTS, and promastigotes treated with CCCP (positive control). FL1-H, rhodamine 123 fluorescence.

Transmission electron microscopy demonstrated that BZTS induced alterations in parasite mitochondria, and we evaluated the ∆Ψm by flow cytometry using Rh 123, which accumulates within energized mitochondria. The histograms (Figure 8) revealed a decrease in total Rh 123 fluorescence intensity, indicating mitochondrial membrane depolarization. BZTS at concentrations of 55, 139, 277, and 555 μM caused 46.2%, 77.3%, 78.5%, and 72.4% decreases in total Rh 123 fluorescence intensity, respectively, compared with the negative control. Such decreases were further quantified as IV values. Promastigotes that were treated with 55, 139, 277, and 555 μM BZTS had IV values of −0.46, −0.77, −0.78, and −0.72, respectively.

### BZTS did not induce phosphatidylserine exposure

**Figure 9:**
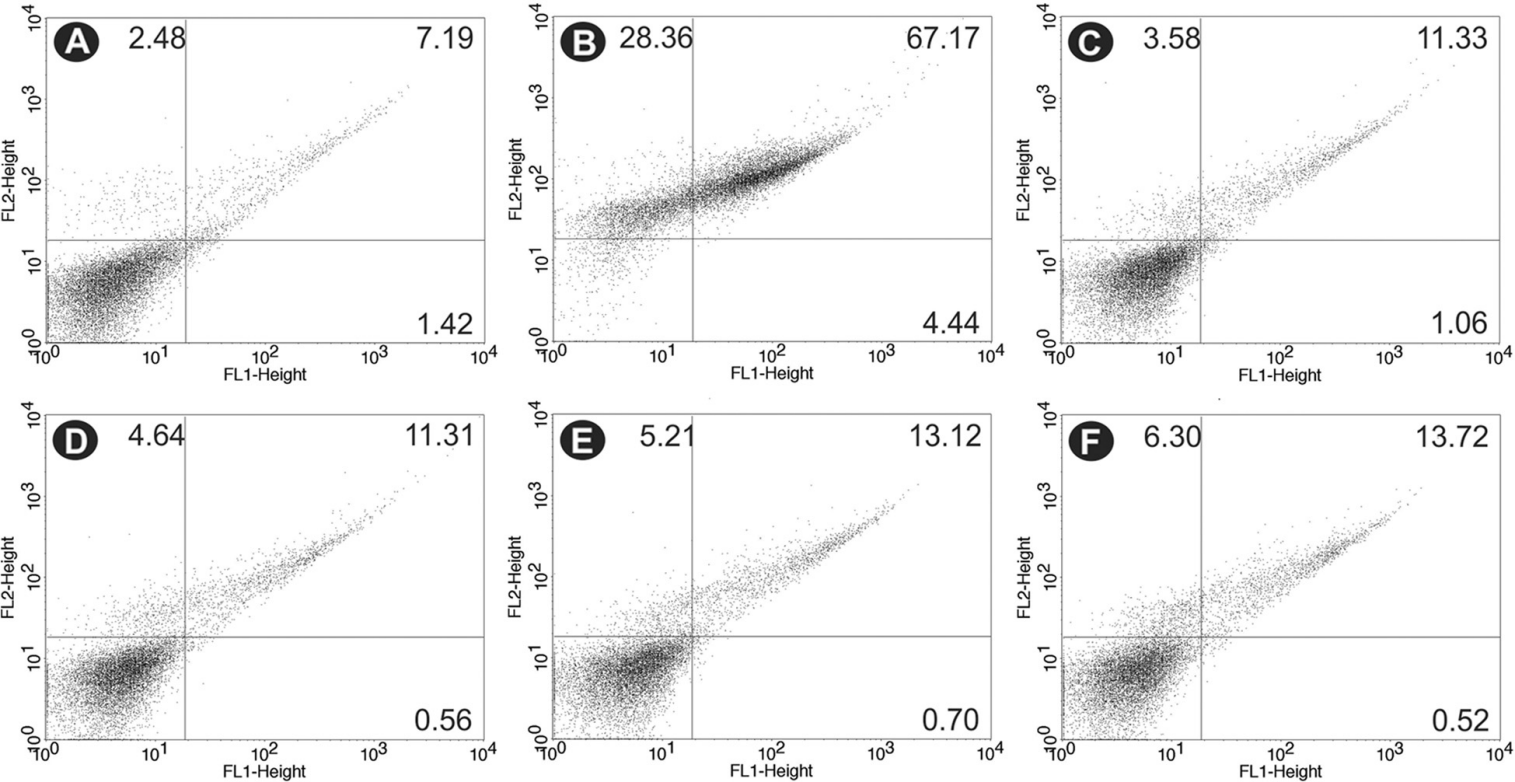
**Phosphatidylserine exposure in untreated** ***Leishmania amazonensis*** **promastigotes (A), promastigotes treated with 100.0 μM CCCP as the positive control (B), and promastigotes treated with 55 μM (C), 139 μM (D), 277 μM (E), and 555 μM (F) BZTS for 24 h using annexin-V FITC and PI.**

Phosphatidylserine (PS) is a phospholipid that is confined to the inner face of the plasma membrane and translocates to the cell surface in apoptotic cells. Annexin V is a calcium-dependent, phospholipid-binding protein that preferentially binds PS (1, 26, 29). Annexin V-FITC was used to evaluate the externalization of phosphatidylserine. As shown in Figure 9, no significant increase in annexin-V fluorescence intensity was observed compared with untreated parasites, indicating no phosphatidylserine exposure. The histograms showed annexin-V fluorescence intensity of 12.4%, 11.9%, 13.9% and 14.2% at BZTS concentrations of 55, 139, 277, and 555 μM, respectively. The negative control showed annexin-V fluorescence intensity of 8.6%. The positive control (CCCP) showed a 71.6% increase in annexin-V fluorescence intensity.

### BZTS caused mitochondrial O_2_^•ˉ^ production

**Figure 10:**
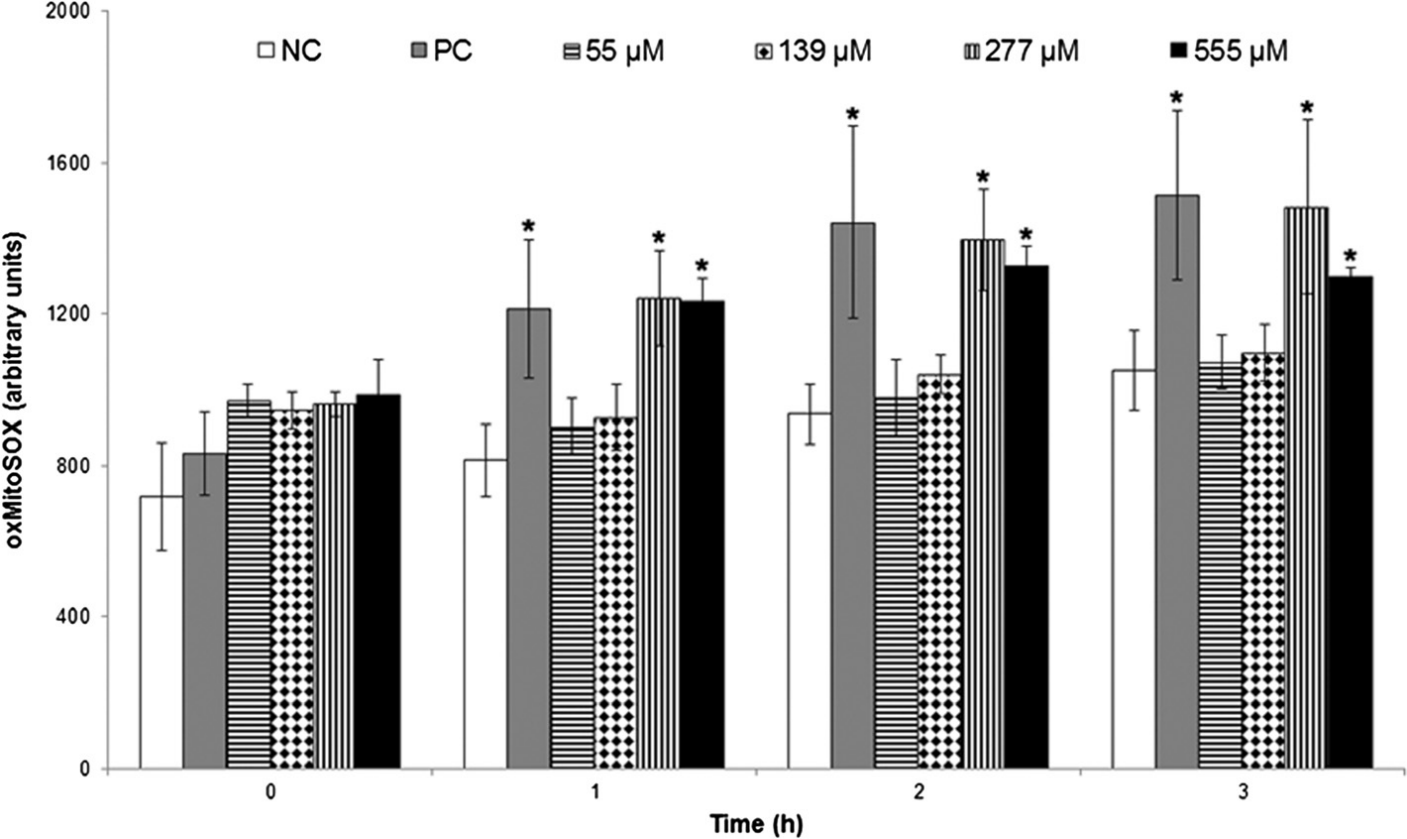
**Mitochondrial O**_**2**_^**−**^ **production in** ***Leishamania amazonensis*** **promastigotes treated with 55, 139, 277, and 555** **μM BZTS for up to 3 h using the fluorescence probe MitoSOX.** Antimycin A (10 μM) was used as a positive control. Fluorescence was measured with a VICTOR X3 spectrofluorometer (Perkin-Elmer). The results are expressed as the mean ± standard error of MitoSOX oxidation (arbitrary units) from three independent experiments. *p ≤ 0.05, significant difference from negative control group.

After observing severe damage in parasite mitochondria and mitochondrial membrane depolarization, we evaluated the production of O_2_^•ˉ^**.** Mitochondrion-derived O_2_^•ˉ^ production was evaluated using MitoSOX reagent, which measures the mitochondrial accumulation of superoxide based on its hydrophobic nature and positively charged triphenylphosphonium moiety. MitoSOX oxidation was higher in BZTS-treated parasites compared with controls. The increase in MitoSOX oxidation in BZTS-treated promastigotes was observed after 1 h of incubation. Treatment with 277 and 555 μM caused higher MitoSOX oxidation than the lower concentrations (Figure 10).

## Discussion

Drug therapy for leishmaniasis has not significantly changed since the beginning of the 20th century, and adequate treatment remains a problem because of toxicity and side effects. New treatments that are more effective and less toxic are urgently needed [16]. Investigations of new drugs with antileishmanial activity, including both natural products and synthetic compounds, have been performed worldwide [17-21].

In the present study, we showed that BZTS had antiproliferative effects on promastigotes and axenic amastigotes, with a reduction of survival of intracellular parasites in macrophages. Moreover, BZTS was more selective for the protozoa than for mammalian cells. BZTS exerted its antileishmanial activity by affecting parasite mitochondrial function, indicated by TEM, variations in ∆Ψm by flow cytometry using Rh 123, and the production of O_2_^•ˉ^ evaluated by MitoSOX oxidation.

The single mitochondrion of the kinetoplastid parasite is an attractive chemotherapeutic target because its functional features are markedly distinct from mammalian mitochondria [22]. Our previous studies showed the *in vitro* antileishmanial activity of benzaldehyde thiosemicarbazone derived from limonene complexed with copper against *L. amazonensis*, with an effect on mitochondrial function [13]. Inácio et al. (2012) reported that epigallocatechin-3-gallate induced death in *L. amazonensis* by directly affecting mitochondrial physiology in treated parasites. These effects were detected as ultrastructural alterations of the mitochondria, organelle injury, and a decrease in Rh 123 fluorescence. Monzote et al. (2014) [23] suggested that the antileishmania activity of the essential oil and major constituents (i.e., ascaridole, carvacol, and caryophllene oxide) of *Chenopodium ambroisiodes* against *L. amazonensis* promastigotes is correlated with mitochondrial dysfunction, reflected by ∆Ψm. Further studies by our group demonstrated ultrastructural mitochondrial alterations and ∆Ψm changes in parasites treated with thiophene derivatives isolated from the aerial parts of *Porophyllum ruderale*, a β-carbolinic compound (*N*-butyl-1-[4-dimethylamino]phenyl-1,2,3,4-tetrahydro-β-carboline-3-carboxamide), and eupomatenoid-5, a neolignan isolated from the leaves of *Piper regnellii* [24-27]. Luque-Ortega et al. (2010) [28] also showed that benzophenone-derived bisphosphonium salts present antileishmania activity against promastigotes of the *Leishmania donovani* causing a dramatically swollen mitochondrion in treated parasites, and a decrease of the electrochemical mitochondrial potential.

Mitochondrial changes may be a consequence of many harmful effects induced by endogenous toxic compounds, such as reactive oxygen species (ROS). BZTS induced the production of these compounds, which may be responsible for mitochondrial injury and the induction of oxidative damage in lipids and proteins, reflected by variations in ∆Ψm using Rh 123 and the production of O_2_^•ˉ^. These results are similar to those reported by Volpato et al. (2013) and Desoti et al. (2012) [29], in which *Trypanosoma cruzi* were treated with *N*-butyl-1-(4-dimethylamino) phenyl-1,2,3,4-tetrahydro-β-carboline-3-carboxamide and (−)-elatol extracted from the red macroalgae *Laurencia dendroidea*, respectively. This effect was also reported by Medina et al. (2012), in which *L. amazonensis* promastigotes were treated with tomatidine.

Electrons move through the mitochondrial respiratory chain during oxidative phosphorylation, and a proton gradient is established across the inner mitochondrial membrane as an energy source for adenosine triphosphate (ATP). A decrease in Rh 123 fluorescence intensity suggests an increase in proton permeability across the inner mitochondrial membrane, which can decrease ATP synthesis and result in parasite death [22,30]. In the present study, we can infer that this process occurred, based on the Rh 123 assays. Furthermore, an association was observed between multivesicular bodies and mitochondrion profiles, likely indicating an autophagic process that removes damaged organelles. In the present study, TEM indicated the presence of several vesicles.

Mitochondrial ultrastructural alterations, the reduction of ΔΨm, and the increase in ROS production caused by BZTS may be explained by calcium release from mitochondria, indicating a direct action of BZTS on the organelle and the inhibition of sterol biosynthesis, leading to alterations in the lipid composition of mitochondrial membranes that can modify mitochondrial function [31,32]. Recently, Shioji Tiuman et al. (2014) [33] demonstrated that when the amastigotes were treated with parthenolide, a lipophilic hydrocarbon compound formed by units of isoprene, revealed mitochondrial damage, suggesting that this compound interferes with the mitochondrial membrane potential leading to alteration of ATP generation and in consequence cell damage takes place.

Another important effect of BZTS on parasites was the accumulation of intracellular lipid bodies in the cytoplasm indicated by fluorescence microscopy. The analysis of the treated parasites using differential interference contrast microscopy revealed alterations in shape, principally by treatment with the IC_90_ of BZTS, showing a rounded shape and evidence of the loss of cytoplasmic content. Similar results were reported by Macedo-Silva et al. (2011), in which promastigote forms were treated with amiodarone, an antiarrhythmic drug used for the treatment of Chagas’ disease. The presence of intracellular lipid bodies may indicate alterations in phospholipids and sterol content [30,34].

BZTS did not significantly increase annexin-V fluorescence intensity in treated parasites, indicating no phosphatidylserine exposure. In contrast, we found that BZTS induced several alterations in the mitochondrial ultrastructure and shape of the parasite. The mitochondria exhibited intense swelling, with the loss of matrix content, the presence of concentric membranes, inner membrane disorganization, severe changes in the mitochondrial membrane potential, the reduction of cell volume, and ROS production. These observations may indicate parasite death caused by apoptosis. Shivahare et al. (2014) [35] showed that a chromenochalcone exhibited leishmanicidal effects against *Leishmania donovani* by causing loss in membrane potential and phosphatidylserine exposure, thus exerting cell death via apoptosis in treated promastigotes.

However, the hypothesis of an autophagic process cannot be discarded because the presence of autophagic vacuoles, increases in the number of lipidic inclusions, and presence of concentric membranes in the cytoplasm are characteristic of autophagic processes and may be associated with the accumulation of aberrant lipids that are probably not entirely incorporated into the plasma membrane or the membranes that line the various cellular organelles [36].

## Conclusion

Our results demonstrated that BZTS has potent antiproliferative activity against different evolutive forms of *Leishmania amazonensis* and induced marked effects on the morphology and ultrastructure of this parasite, such as interference with various cellular processes that led to changes in shape and mitochondrial function, the accumulation of lipid droplets, and features found in cells that die from apoptotic or autophagic processes.

## Methods

### Chemistry

All of the melting points were determined using a Microquímica model MQAPF-301 apparatus. Infrared spectra were obtained using KBr pellets in an FT-IR BOMEM spectrophotometer. Nuclear magnetic resonance (^1^H NMR and ^13^C NMR) spectra were obtained using a Varian Mercury Spectrometer (300 MHz for ^1^H and 75.5 MHz for ^13^C) in CDCl_3_/TMS solution at 298°C, with chemical shifts (δ) given in parts per million. Optical rotation was determined using a Perkin Elmer Model 343 polarimeter at 20°C and 589 nm, with chloroform as the solvent.

### Synthesis of 4-nitrobenzaldehyde thiosemicarbazone derived from S-limonene

For the synthesis of 4-nitrobenzaldehyde thiosemicarbazone, a drop of solution of 5% sulfuric acid and 1.0 mmol (227.4 mg) thiosemicarbazide derived from *S*-limonene were added to a flask that contained 1.0 mmol (151.2 mg) 4-nitrobenzaldehyde solubilized in ethanol (30 ml) (Yamaguchi et al., 2009) [37]. The mixture was stirred at room temperature for 30 min, and the progression of the reaction was monitored by TLC. The solvent was evaporated under reduced pressure, and the crude product was recrystallized from a mixture of ethanol:chloroform (1:3) to yield yellow crystals: yield (93%); melting point (165.4°C); optical rotation (α^20^_D_): −30.0° [IV (KBr, υ _max_): 3325 (NH); 3291 (NH); 2990–2900 (CH); 1544 (C = S); 1373 (C = N). ^1^H NMR (300 MHz, CDCl_3_): δ = 1.57 (s, 3H, H8’); 1.60 (s, 3H, H9’); 1.67 (s, 3H, H10’); 1.86 (m, 2H, H6’); 2.04 (m, 2H, H2’); 2.07 (m, 2H, H5’); 2.69 (m, 1H, H1’); 5.40 (m, 1H, H3’); 7.56 (sl, 1H, H4); 7.75 (dd, *J* = 8.7; 4.2 Hz, 2H, H3”/H7”); 7.92 (s, 1H, H1”); 8.27 (dd, *J* = 8.7; 4.0 Hz, 2H, H4”/H6”); 9.83 (sl, 1H, H2); ^13^C NMR (75.5 MHz, CDCl_3_): δ = 23.3 (C10’); 23.9 (C9’); 24.1 (C8’); 24.2 (C6’); 26.5 (C2’); 31.0 (C5’); 40.8 (C1’); 59.1 (C7’); 120.2 (C3’); 124.1 (C4”/C6”); 127.5 (C3”/C7”); 134.1 (C4’); 138.5 (C1”); 139.7 (C2”); 148.2 (C5”); 175.1 (C3)] (Figure 1).

### Parasite culture

*Leishmania amazonensis* promastigotes (MHOM/BR/Josefa) were maintained at 25°C in Warren’s medium [brain-heart infusion plus hemin (10 μg/mL) and folic acid (10 μg/mL), pH 7.2] supplemented with 10% heat-inactivated fetal bovine serum (FBS; Gibco Invitrogen, New York, NY, USA). Axenic amastigotes were obtained by *in vitro* transformation of infective promastigotes by a progressive temperature increase and pH decrease. These forms were maintained in Schneider’s medium (Sigma, St. Louis, MO, USA), pH 4.6, that contained 20% FBS at 32°C.

### *In vitro* antiproliferative assay

Promastigotes in the logarithmic phase (1 × 10^6^ parasites/ml) were grown in 24-well culture microplates at 25°C in Warren’s medium supplemented with 10% FBS, and axenic amastigote forms (1 × 10^6^ parasites/ml) were grown in 12-well culture microplates at 32°C in Schneider’s medium supplemented with 20% FBS. The parasites were incubated in the presence or absence of 2.7, 13.9, 27.7, 139, and 277 μM BZTS and incubated for 72 h. Dimethyl sulfoxide (DMSO) was used to solubilize the stock solution of the compound. The final DMSO concentration did not exceed 1%, which has no deleterious effects on the parasites. Antileishmania activity was determined by directly counting free-living parasites in a Neubauer chamber, and the concentration that inhibited growth by 50% (IC_50_) was determined graphically by plotting the concentration *vs*. percentage growth inhibition.

### Activity against intracellular amastigotes

Peritoneal macrophages were collected from BALB/c mice by washing with cold phosphate-buffered saline (PBS) supplemented with 3% FBS (The protocol was approved by the Ethical Committee of the State University of Maringa. Approval no. 074/2011). Sterile glass coverslips were placed in the wells of a 24-well microplate, and 5 × 10^5^ cells/ml were added to each well in RPMI 1640 medium supplemented with 10% FBS. The microplate was incubated for 2 h at 37°C in a 5% CO_2_-air mixture to adhere macrophages. The macrophage monolayer was infected with promastigote forms at a 7:1 parasite:macrophage ratio. After 4 h of interaction at 34°C in a 5% CO_2_-air mixture, the microplate was washed with RPMI 1640 medium to remove the non-interiorized parasites. Afterward, the infected macrophages were treated with BZTS at concentrations of 2.7, 5.5, 13.9, and 27.7 μM and incubated for 48 h. The percentage of infected macrophages was evaluated after Giemsa staining by microscopically counting the number of amastigotes per macrophage.

### *In vitro* cytotoxicity assay

The J774A1 macrophage cell monolayer was suspended to yield 5 × 10^5^ cells/ml in RPMI 1640 medium supplemented with 10% FBS and added to each well in 96-well microtiter plates. The plates were incubated in a 5% CO_2_-air mixture at 37°C to obtain confluent cell growth. The macrophage monolayer was treated with different concentrations of BZTS (2.7, 13.9, 27.7, 139, and 277 μM) for 48 h. After treatment, the medium was removed, and the macrophage monolayer was washed with PBS, and 50 μL of MTT (3-[4,5-dimethylthiazol-2-yl]-2,5-diphenyltetrazolium bromide formazan; 2 mg/mL) was added. The microplate was then incubated for 4 h in a 5% CO_2_-air mixture at 37°C. After the incubation period, 150 μl DMSO was added, and the microplate was homogenized. Absorbance was read in a microplate reader (BIO-TEK Power Wave XS) at 570 nm. The percentage of viable macrophages was calculated compared with controls (i.e., macrophages cultured in medium without drug). The 50% cytotoxic concentration (CC_50_) was determined by logarithm regression analysis of the data.

### Electron microscopy

#### Scanning electron microscopy

For the morphological analysis, promastigotes that were treated for 72 h at 25°C with concentrations that corresponded to the IC_50_ and IC_90_ for BZTS were fixed in 2.5% glutaraldehyde in 0.1 M sodium cacodylate buffer for 1–3 h. Subsequently, parasites were adhered on poly-L-lysine-coated coverslips and dehydrated in an ascending series of ethanol. The samples were critical-point-dried in CO_2_, coated with gold, and observed in a Shimadzu SS-550 scanning electron microscope.

#### Transmission electron microscopy

Ultrastructural analysis was performed with promastigote and axenic amastigote forms that were treated for 72 h at 25°C and 32°C with concentrations that corresponded to the IC_50_ and IC_90_ for BZTS, respectively. After washing with PBS, the parasites were fixed in 2.5% glutaraldehyde in 0.1 M sodium cacodylate buffer at 4°C and post-fixed in a solution that contained 1% osmium tetroxide, 0.8% potassium ferrocyanide, and 5 mM calcium chloride. The parasites were dehydrated in an acetone series and embedded in Epon resin for 72 h at 60°C. Ultrathin sections were stained with 5% uranyl acetate and lead citrate and examined in a JEOL JEM 1400 transmission electron microscope.

### Flow cytometry

#### Evaluation of cell membrane integrity

Promastigotes were exposed to 55, 139, 277, and 555 μM BZTS for 24 h at 25°C and then harvested and washed with PBS. The cells were incubated with 50 μl of 2 mg/ml propidium iodide (PI) for 5 min according to the manufacturer’s instructions. Immediately thereafter, the cells were analyzed using a BD FACSCalibur flow cytometer equipped with CellQuest Pro software. A total of 10,000 events were acquired in the region that corresponded to the parasites. Alterations in PI fluorescence were determined compared with untreated parasites. Digitonine (40.0 μM) was used as a positive control.

#### Determination of mitochondrial transmembrane potential (∆Ψm)

Promastigotes were exposed to 55, 139, 277, and 555 μM BZTS for 24 h at 25°C. The cells were then incubated with 1 μl (5 mg/ml in ethanol) of rhodamine 123 (Rh 123; Sigma-Aldrich, St. Louis, MO, USA) for 15 min, resuspended in 0.5 ml PBS, and incubated for an additional 30 min. The assay was conducted according to the manufacturer’s instructions. The parasites were analyzed using a BD FACSCalibur flow cytometer and CellQuest Pro software. A total of 10,000 events were acquired in the region that corresponded to the parasites. Carbonyl cyanide 3-chlorophenylhydrazone (CCCP; 100.0 μM) was used as a positive control. Alterations in Rh 123 fluorescence were quantified using an index of variation (IV) obtained from the equation *(Mt - Mc)/Mc*, in which Mt is the median fluorescence for the BZTS-treated parasites, and Mc is the median fluorescence for the untreated parasites. Negative IV values correspond to depolarization of the mitochondrial membrane.

#### Detection of phosphatidylserine exposure by annexin-V-FITC labeling

Promastigotes were treated with BZTS (55, 139, 277 and 555 μM) for 24 h at 25°C and then harvested and washed with PBS. The cells were then resuspended in 500 μl of binding buffer (140 mM NaCl, 5 mM CaCl_2_, and 10 mM HEPES-Na, pH 4.7), followed by the addition of 5 μl of FITC-conjugated annexin V. The reaction was incubated for 5 min at room temperature. Afterward, 400 μl of binding buffer and 50 μl PI were added. Fluorescence was measured using a BD FACSCalibur flow cytometer and CellQuest Pro software. A total of 10,000 events were acquired in the region that corresponded to the parasites as described above. CCCP (100.0 μM) was included as a positive control, and untreated parasites served as the negative control. The parasites were considered necrotic when they were PI-positive and apoptotic when stained with annexin-V (PI-positive or -negative).

### Determination of cell volume of parasites

Promastigotes were treated with BZTS (55, 139, 277, and 555 μM) for 24 h at 25°C and then harvested and washed with PBS. The parasites were then analyzed using a BD FACSCalibur flow cytometer and CellQuest Pro software. Density plots of forward (FSC) *vs*. side (SSC) scatter represent the acquisition of 10,000 events.

### Fluorimetric detection of mitochondrial superoxide anion radical (O_2_^•ˉ^)

Promastigotes were harvested and washed with Krebs-Henseleit (KH) solution buffer, pH 7.3, that contained 15 mM NaHCO_3_, 5 mM KCl, 120 mM NaCl, and 0.7 and 1.5 mM NaH_2_PO_4_. The cells were loaded with 2.0 ml of 5 μM MitoSOX reagent (3,8-phenanthridinediamine-5-[6-triphenylphosphoniumhexyl]-5,6-dihydro-6-phenyl). The parasites were incubated for 10 min at 37°C and protected from light. After incubation with MitoSOX reagent, the parasites were washed three times with KH buffer and treated with 55, 139, 277, and 555 μM BZTS. Antimycin A (10 μM) was used as a positive control. MitoSOX detection was performed using black 96-well plates for 3 h. Fluorescence was measured at excitation and emission wavelengths of 510 and 580 nm, respectively, in a VICTOR X3 spectrofluorometer (Perkin Elmer). The results are expressed as arbitrary units of MitoSOX.

### Detection of cytoplasmic lipid bodies by nile red staining

Promastigote forms of *L. amazonensis* were treated with concentrations that corresponded to the IC_50_ and IC_90_ for BZTS as described previously in an antiproliferative assay. The treated parasites were then harvested, washed twice in PBS, and directly stained with 10 μg/ml Nile Red (Sigma-Aldrich, St. Louis, MO, USA) for 30 min at room temperature. The cytoplasmic lipid bodies in the parasites were detected with an epifluorescence microscope (Olympus BX51) equipped with a WG filter. The parasites were photographed using an Olympus UC30 camera.

### Statistical analysis

In the cellular experiments, the 50% growth inhibition value (IC_50_) was determined from the linear concentration-response curves, and the results are expressed as the mean and standard deviation of at least three independent experiments. Nonparametric data were analyzed using one-way analysis of variance (ANOVA), and significant intergroup differences were analyzed using Tukey’s test. All of the statistical analyses were performed at the *p* < 0.05 level of significance.

## Additional files

Additional file 1:
**Graphical 1.** Inhibition percentage of promastigote and axenic amastigote forms of *Leishmania amazonensis* treated with BZTS for 72 h. The data are expressed as the means from three independent tests. Additional file 2:
**Graphical 2.** Inhibition percentage of intracellular amastigote forms of *Leishmania amazonensis* treated with BZTS for 48 h. The data are expressed as the means from three independent tests. Additional file 3:
**Graphical 3.** Inhibition percentage of J774A1 macrophages after treatment with BZTS for 48 h. The data are expressed as the means from three independent tests.
