# Supplementary figures and images for: Cell death and ultrastructural alterations in Leishmania amazonensis caused by new compound 4-Nitrobenzaldehyde thiosemicarbazone derived from S-limonene
Source: BMC Microbiol. 2014 Sep 26;14:236. doi: 10.1186/s12866-014-0236-0 (PMC4188478; doi:10.1186/s12866-014-0236-0)

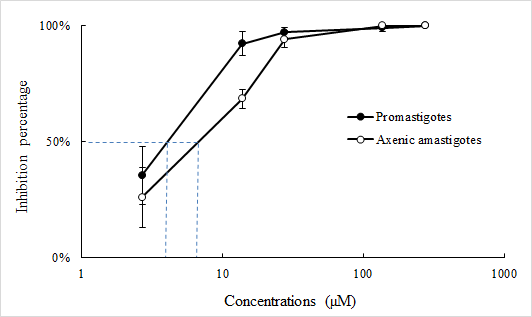

Supplement: Additional file 1: — Graphical 1. Inhibition percentage of promastigote and axenic amastigote forms of Leishmania amazonensis treated with BZTS for 72 h. The data are expressed as the means from three independent tests. [file 12866_2014_236_MOESM1_ESM.tiff]

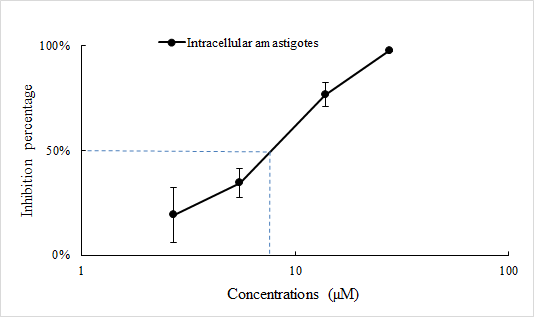

Supplement: Additional file 2: — Graphical 2. Inhibition percentage of intracellular amastigote forms of Leishmania amazonensis treated with BZTS for 48 h. The data are expressed as the means from three independent tests. [file 12866_2014_236_MOESM2_ESM.tiff]

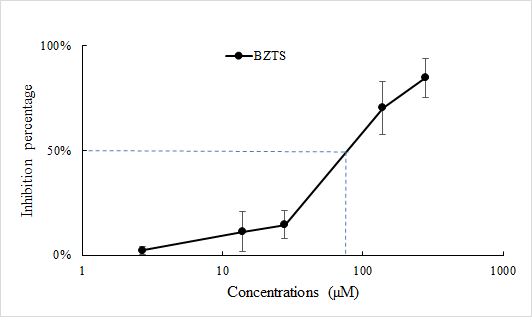

Supplement: Additional file 3: — Graphical 3. Inhibition percentage of J774A1 macrophages after treatment with BZTS for 48 h. The data are expressed as the means from three independent tests. [file 12866_2014_236_MOESM3_ESM.tiff]
